# Effect of the Inoculation Site of Bovine and Avian Purified Protein Derivatives (PPDs) on the Performance of the Intradermal Tuberculin Test in Goats From Tuberculosis-Free and Infected Herds

**DOI:** 10.3389/fvets.2021.722825

**Published:** 2021-08-27

**Authors:** Javier Ortega, Álvaro Roy, Julio Álvarez, José Sánchez-Cesteros, Beatriz Romero, Jose A. Infantes-Lorenzo, José L. Sáez, Marisol López, Lucas Domínguez, Lucia de Juan, Javier Bezos

**Affiliations:** ^1^VISAVET Health Surveillance Centre, Complutense University of Madrid, Madrid, Spain; ^2^Departamento de Sanidad Animal, Facultad de Veterinaria, Universidad Complutense de Madrid, Madrid, Spain; ^3^Servicio de Inmunología Microbiana, Centro Nacional de Microbiología, Instituto de Investigación Carlos III, Majadahonda, Spain; ^4^Ministerio de Agricultura, Pesca y Alimentación, Madrid, Spain; ^5^Servicio Territorial de Agricultura, Ganadería y Desarrollo Rural de Ávila- Sección de Sanidad y Producción Animal, Ávila, Spain

**Keywords:** tuberculosis, goat, intradermal tests, inoculation site, harmonization

## Abstract

The single and comparative intradermal tuberculin (SIT and CIT) tests are used for the ante-mortem diagnosis of caprine tuberculosis (TB). The tuberculin injection site has been associated with a different performance of the test in cattle. In contrast to that required in cattle in Europe (cervical injection), it can be carried out in the scapular region in goats. Nevertheless, there are no previous data concerning the effect of the injection site on the performance of the test in goats. The aim of the present study was to evaluate the effect of two different inoculation sites (cervical and scapular) on the performance of the SIT/CIT tests. This was done by intradermally inoculating 309 goats from two infected herds and one TB-free herd with both avian and bovine PPDs in the mid-cervical and scapular regions. None of the animals from the TB-free herd had positive reactions, and the number of reactors was not significantly higher, regardless of the inoculation site, in the high and low prevalence herds. However, significantly higher increases in skin fold thickness were observed on the cervical site when compared to the scapular site after the avian and bovine PPD inoculations in the TB-free herd (*p* < 0.001) and after the bovine PPD injection in the high prevalence herd (*p* = 0.003). The presence of clinical signs was also more evident on the cervical site when using avian and bovine PPDs in the high prevalence herd (*p* < 0.01). In contrast, increases in higher skin fold thickness were observed on the scapular site when compared to the cervical site after the bovine and avian PPD inoculations were employed in the low prevalence herd (*p* < 0.01). These results suggest that the cervical injection of PPDs may improve the sensitivity of the intradermal tuberculin test in high TB prevalence caprine herds, mainly owing to the increased presence of local clinical signs and a better performance of the CIT test. Moreover, specificity was not affected when using standard interpretations, although further analyses in a great number of herds are required in order to confirm these findings.

## Introduction

Tuberculosis (TB) is a zoonotic infection disease caused by members of the *Mycobacterium tuberculosis* complex (MTBC) (1) that affects humans and a wide range of wild and domestic animals (2–4). Caprine TB is caused principally by *Mycobacterium bovis* and *Mycobacterium caprae* (5, 6), with the latter being the main etiological agent in Spain (7). Several studies have demonstrated the role played by goats in the transmission and maintenance of TB in different species (3, 8). The Spanish bovine TB eradication programme recommends the testing of goats that have any epidemiological link with cattle (9). Moreover, certain regions of Spain carry out specific TB control programmes on caprine herds. As occurs with the bovine TB eradication programme, TB eradication programmes in goats are based principally on a test and cull strategy carried out using the single and comparative intradermal tuberculin (SIT and CIT) tests and slaughterhouse surveillance (10). The interferon-gamma release assay (IGRA), which is an official ancillary test for TB diagnosis in cattle, is not, however, routinely used with goats (10–13).

The SIT and CIT tests are considered to be valuable diagnostic tools, although their performance in terms of sensitivity and specificity can be affected by several factors (14). These factors include the period between consecutive tests (15), co-infections with other non-tuberculous mycobacteria (16), vaccination against *Mycobacterium avium* subsp. *paratuberculosis* (MAP) (17, 18), the interpretation criteria applied (6), the potency of purified protein derivatives **(**PPDs) (19), and the correct use and maintenance of injection syringes (20, 21). Individual factors such as breed (22), age (23), and host susceptibility to *M. bovis* infection (24, 25) may additionally affect the performance of the test and should be taken into account.

The effect of the tuberculin inoculation site on the performance of the test has been studied in cattle (26–28), camelids (29), and deer (30). Two sites have historically been used for PPD inoculation in cattle: the neck and the caudal fold. The caudal fold is used in several countries, such as the United States or New Zealand (31, 32), although a lower sensitivity has been observed when using this location in comparison to the cervical region (33, 34). The neck (cervical region) is, therefore, the recommended inoculation site within the European Union (Regulation EU 2016/429 and Commission Delegated Regulation EU 2020/688). However, the sensitivity of the cervical test may vary depending on the exact location in which the PPD is injected, as demonstrated by Casal et al. (28), who concluded that the PPD injection in the anterior neck area in cattle maximizes sensitivity without compromising specificity when compared to scapular area (28).

The vast majority of studies carried out with goats have reported the use of the cervical test, although the effect of the inoculation site has not been evaluated for this species (13). However, certain regional TB caprine eradication programmes include the scapular region as the recommended location in which to carry out the SIT and CIT tests (e.g., Orden AYG/415/2016), probably owing to the greater ease of performance when compared to the neck. The aim of the present study was, therefore, to carry out the first documented evaluation of whether the PPD inoculation site has an effect on the performance of the SIT and CIT tests when used with goats.

## Materials and Methods

### Study Design

The study was performed with two TB-infected Guadarrama–breed dairy herds and one TB-free Murciano-granadina–breed dairy herd located in central Spain. Both TB-infected herds had previously been confirmed by means of bacteriological culture (*M. bovis* SB0121). This had led to their classification as high (*n* = 158; apparent prevalence of 72.2%, 95% CI 70.0–81.2; range of study population: 1–3 years old) and low (*n* = 193; apparent prevalence of 7.3%, 95% CI 4.4–11.8; range of study population: 3–5 years old) prevalence herds on the basis of previous TB testing using the SIT test and IGRA in parallel. The TB-free herd (n = 244; range of study population: 1–5 years old) had no previous history of TB and had been tested annually for more than 10 years within the framework of a regional eradication programme. Moreover, a vaccination programme against MAP had been employed with these three herds, using the Gudair vaccine (CZ Vaccines, Porriño, Spain) in goats under 6 months of age.

Ninety-six adult goats were randomly selected from the high prevalence herd, 94 from the low prevalence herd, and 119 from the TB-free herd, and all animals were then subjected to the intradermal tests and IGRA. The intradermal tuberculin tests were carried out using two different inoculation sites (cervical and scapular) for both PPDs in all three herds. Serum samples were also assayed for TB by using an indirect ELISA that detects antibodies against a protein complex purified from bovine PPD (CZ Vaccines, Porriño, Spain), denominated as P22 (P22 ELISA) (35). IGRA and P22 ELISA were performed using blood samples collected just before the PPD injection.

The animals included in the study were not experimental animals. All handling and sampling procedures were carried out in accordance with local and Spanish legislation (Royal Decree 727/2011).

### Intradermal Tuberculin Tests

**Figure 1:**
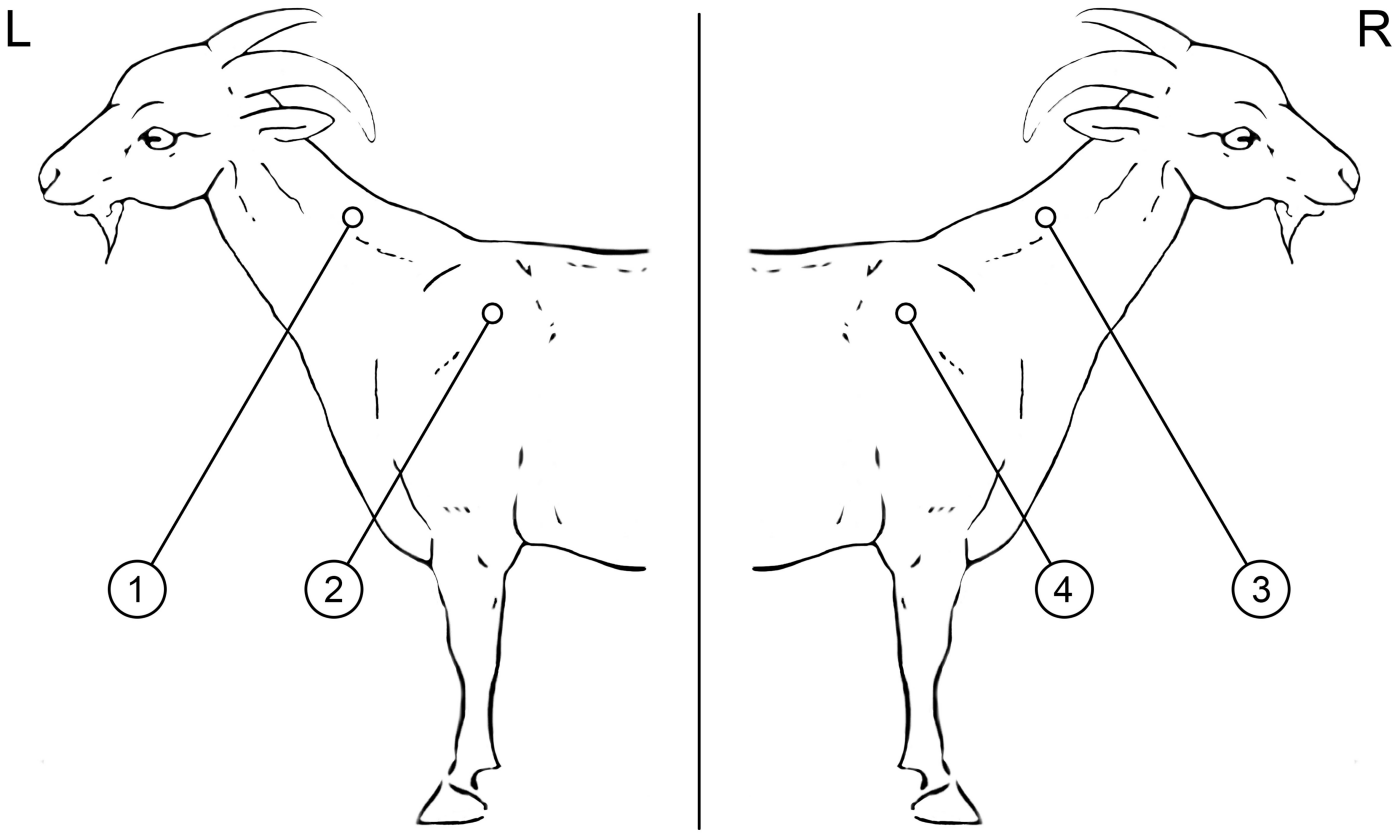
Location of bovine and avian PPD injection sites. (L) **Left** side: (1) Bovine PPD cervical, (2) Bovine PPD scapular; (R) **Right** side: (3) Avian PPD cervical, (4) Avian PPD scapular.

The SIT and CIT tests were performed in the mid-cervical and scapular regions on both sides of each animal (Figure 1) using a Dermojet syringe (Akra Dermojet, Pau, France), and the reactions were interpreted 72 h later by the same veterinarian that had performed the tests. All animals received two bovine PPD (left side, cervical, and scapular) and two avian PPD (right side, cervical and scapular) intradermal injections of 0.1 ml (CZ Vaccines, Porriño, Spain). All the tests were performed in accordance with the protocol published by the European Reference Laboratory (EU-RL) for bovine TB following Regulation EU 2016/429 and Commission Delegated Regulation EU 2020/688 and Royal Decree 2611/1996, as described previously (36). Two cut-off points were applied in this study. When using the standard interpretation, an increase in skin fold thickness of 4 or more mm or the presence of clinical signs (exudation, oedema, pain, or necrosis) were considered a positive reaction to the SIT test. When using the severe interpretation, a skin-fold thickness of >2 mm or the presence of clinical signs were considered positive. An animal was considered to be positive to the CIT test if the bovine reaction was greater than the avian reaction by more than 4 mm, or there were clinical signs on the bovine PPD inoculation site (standard interpretation). When using the severe interpretation, positive reactors to the CIT test were those that had a bovine reaction of more than 2 mm that was greater than the avian reaction, or in which the presence of clinical signs was observed on the bovine PPD inoculation site. The same interpretation criteria were used for both the cervical and scapular inoculation sites.

### Interferon-Gamma Release Assay (IGRA)

Blood samples were collected from the jugular vein using lithium heparin tubes (BD Vacutainer Becton, Dickinson and Company, Franklin Lakes, USA) and stimulated with bovine and avian PPDs (CZ Vaccines, Porriño, Spain) at a final concentration of 20 μg/mL for the detection of IFN-γ production. The blood samples were then processed as described previously (12). IFN-γ production was measured using a commercial IGRA (Bovigam TB kit, Thermo Fisher Scientific, Waltham, USA) according to the manufacturer's instructions, and the results were interpreted as described elsewhere (37), using the cut-off points of 0.05 and 0.1 as severe or standard intepretations, respectively, according to the protocol established by the EU-RL for bovine TB.

### P22 ELISA

Blood samples were collected from the jugular vein using plastic serum tubes (BD Vacutainer Becton, Dickinson and Company, Franklin Lakes, USA). Serum samples were stored at room temperature for 24 h and then centrifuged for 15 min at 650 g and conserved at −20°C until the assay. The ELISA was performed as described previously (38). The sample results were expressed as an ELISA percentage (E%), which was calculated using the following formula:

$$\begin{array}{l} \text{Sample E}\%\text{= }[(\text{mean sample OD/}(\text{2}\times \text{mean of negative control }\\ \text{ OD})]\times \text{100} \end{array}$$

Serum samples with E% values >150 or 100 were considered positive to the standard and severe interpretations, respectively.

### Statistical Analysis

All the statistical tests were carried out using SPSS Statistics 25 (IBM, New York, USA) and R version 4.0.5 software (39) and interpreted by considering that a *p*-value of 0.05 was indicative of statistical significance. Wilson's 95% confidence intervals (CI) were calculated for the percentage of animals found positive to the different tests. Cohen's kappa coefficient (κ) was used to calculate the agreement between tests (intradermal tests, IGRA and P22 ELISA) and inoculation sites (cervical and scapular). The Kappa statistic (*k*) was interpreted as follows: ≤ 0 poor, 0.01–0.20 slight, 0.21–0.40 fair, 0.41–0.60 moderate, 0.61–0.80 substantial, and 0.81–1.00 almost perfect agreement. Quantitative differences in the skin fold thickness (expressed in mm), IFN-y levels (OD), and ELISA percentage (E%) within a herd were analyzed using the Wilcoxon signed-rank test. The proportion of reactors to the cervical and scapular inoculation sites within a herd was compared using McNemar's test. The proportions of test reactors in each herd were compared using Fisher's exact test. The skin fold thickness, IFN-γ OD, and ELISA E% obtained for each herd were analyzed employing the Kruskal–Wallis and Mann–Whitney U tests.

## Results

### High Prevalence Herd

**Figure 2:**
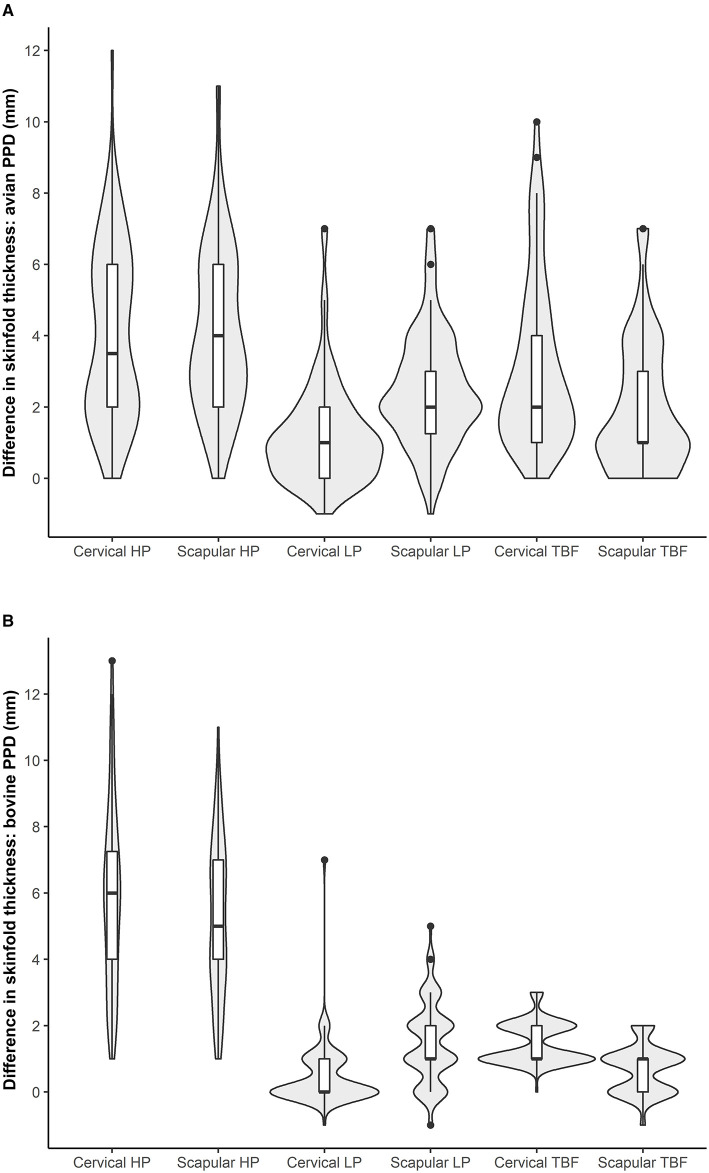
Distribution of difference in skinfold thickness after avian **(A)** and bovine **(B)** PDD injection on cervical and scapular sites in high prevalence (HP), low prevalence (LP), and TB-free (TBF) herds. The frequency of skinfold thickness measures observed in each group is correlated with the curves of the violin plot.

The median increases in skin fold thickness observed after the bovine PPD injection on the cervical and scapular sites were 6 mm (IQR = 4–7.75 mm) and 5 mm (IQR = 4–7 mm), respectively, and the differences observed were significant (*p* = 0.003; Figure 2B). No differences between the two inoculation sites were observed as regards an increase in skin fold thickness when using the avian PPD (*p* = 0.372; Figure 2A). With regard to the qualitative results obtained for this herd, 84/96 (87.5%) and 91/96 (94.8%) goats were positive reactors to the SIT test on at least one inoculation site when using the standard and severe interpretation, respectively. However, there were no differences in the number of reactors to the SIT test as regards either the cervical or the scapular inoculation sites when using both the severe and standard interpretations (*p* > 0.05, Table 1). In this herd, 65 (67.7%) and 75 (78.1%) goats had positive reactions in both locations (cervical and scapular) when using the standard and severe SIT test interpretations, respectively. The agreement between both locations was fair to moderate when using the severe and standard interpretations (*k* = 0.29 and *k* = 0.43, respectively).

**Table 1:** Number and percentage of reactors to the SIT and CIT tests (on cervical and scapular sites), IGRA, and P22 ELISA observed in the caprine herds studied herein.

**Herd**	**n**	**SIT test^a^**				**CIT test^b^**							
		**Cervical standard**	**Cervical severe**	**Scapular standard**	**Scapular severe**	**Cervical standard**	**Cervical severe**	**Scapular standard**	**Scapular severe**	**IGRA 0.05^c^**	**IGRA 0.1^c^**	**P22 ELISA 100^d^**	**P22 ELISA 150^d^**
High prevalence	96	76 (79.2%)	84 (87.5%)	73 (76%)	82 (85.4%)	29 (30.2%)	38 (39.6%)	12 (12.5%)	24 (25%)	49 (51%)	37 (38.5%)	78 (81.2%)	69 (71.8%)
Low prevalence	94	1 (1.1%)	1 (1.1%)	5 (5.3%)	17 (18.1%)	1 (1.1%)	1 (1.1%)	0	1 (1.1%)	6 (6.4%)	3 (3.2%)	31 (32.9%)	7 (7.4%)
TB-free	119	0	7 (5.9%)	0	0	0	0	0	0	3 (2.5%)	0	25 (21%)	10 (8.4%)

a *An animal was considered to be a positive reactor to the SIT test when there was an increase ≥ 4 mm (standard interpretation) or >2 mm (severe interpretation) in the skinfold thickness and/or the presence of clinical signs were observed*.

b *An animal was considered to be a positive reactor to the CIT test when the bovine reaction was greater than the avian reaction by more than 4 mm (standard interpretation) or the bovine reaction was ≥3 mm and greater than the avian reaction (severe interpretation); and/or there were clinical signs on the bovine PPD inoculation site*.

c *An animal was considered to be positive to the IGRA if the optical density (OD) of a sample stimulated with bovine PPD minus the OD of PBS was >0.05 (standard interpretation) or 0.1 (severe interpretation) and greater than the OD of the sample stimulated with avian PPD*.

d *An animal was considered to be positive to the P22 ELISA when the E(%) value was >150 (standard interpretation) or 100 (severe interpretation)*.

In contrast to the SIT test results, a significantly higher number of reactors to the CIT test was observed as regards the cervical site in comparison to the scapular site when using the standard interpretation (*p* < 0.01) (Table 1). The agreement between the number of reactors to the CIT test when administered on the scapular and cervical sites was moderate upon using the standard (*k* = 0.45) and severe (*k* = 0.44) interpretations.

Of those animals that obtained positive results to the SIT and CIT tests, seven had local clinical signs after the bovine PPD injection on the cervical site, and only one on the scapular site (*p* < 0.01). A higher number of goats with clinical signs on the cervical site when compared to the scapular site were similarly observed after the avian PPD injection (*p* < 0.01).

With regard to the *in vitro* techniques, the P22 ELISA detected a higher number of skin-test positive animals than did IGRA (Table 1). When the SIT test was interpreted in parallel with IGRA cut-off point of 0.05, 86 (89.6%) and 91 (94.8%) of 96 animals were considered infected when using the standard and severe interpretations of the SIT test, respectively. These proportions increased to 96.8% and 98.9% when applying the combination of the P22 ELISA (E% 100) and the standard and severe interpretations of the SIT test, respectively.

### Low Prevalence Herd

A high proportion of animals in the low prevalence herd (61/94) did not undergo any increase in skin fold thickness on the cervical site when using the bovine PPD (median = 0 mm, IQR 0–1 mm; Figure 2B). However, unlike that reported in the high prevalence herd, significantly higher increases in skin fold thickness (*p* < 0.01) were observed on the scapular site in comparison to the cervical site after the bovine and avian PPD inoculations (Figure 2A). Differences in the proportion of reactors according to the inoculation site (cervical/scapular) were observed only when using the severe interpretation of the SIT test (*p* < 0.01, Table 1) but not with the severe interpretation of the CIT test (*p* > 0.05). No local clinical signs were detected in this herd, regardless of the PPD (bovine or avian) or the inoculation site (cervical or scapular) used.

Similarly to that which occurred with the high prevalence herd, a higher number of reactors were detected when using the P22 ELISA in comparison to IGRA (*p* < 0.01, Table 1). The use of IGRA (0.05 cut-off point) and the P22 ELISA (E% 100) as ancillary techniques to the severe SIT test increased the proportion of positive animals to 23.4 and 43.6%, respectively. None of the animals that reacted to the SIT test on the scapular site were positive to IGRA, and only 1 and 5 reactors to the standard and severe interpretation of the SIT test were positive to the P22 ELISA (E% 100).

### TB-Free Herd

In the TB-free herd, 66 out of 119 of the animals had a median increase in skin fold thickness of 1 mm on the cervical (IQR = 1–2 mm) and scapular sites (IQR = 0–1 mm) when using the bovine PPD (Figure 2B). The median increase in skin fold thickness was, meanwhile, 2 mm (median = 2 mm, IQR = 1–4 mm) and 1 mm (median = 1 mm, IQR = 1–3 mm) on the cervical and scapular sites, respectively, when using the avian PPD (Figure 2A). No positive reactors were detected when using the standard interpretation of the SIT and CIT tests, regardless of the inoculation site (Table 1). Seven animals (5.9%) from this herd underwent an increase in skin fold thickness of 3 mm on the cervical site after the bovine PPD injection and were, therefore, positive to the severe SIT test, whereas no positives were observed in the case of the scapular site (Table 1, *p* = 0.016). No reactors to the severe CIT test were, however, detected for any of the inoculation sites (Table 1). No clinical signs were detected in this herd when using the bovine or avian PPD, regardless of the inoculation site (cervical or scapular).

In addition to the seven animals that were positive to the severe SIT test on the cervical site, another three different animals were positive to IGRA when using the severe cut-off point (0.05). When interpreting the P22 ELISA in combination with the IGRA (0.05 cut-off), the percentage of positive reactors increased significantly (23.5% and 11%, E% 100 and 150 cut-off points, respectively, *p* < 0.01, Table 1). None of the seven animals that was positive as regards the administration of the severe SIT test to the cervical site was positive to IGRA or the P22 ELISA, regardless of the cut-off point.

## Discussion

The present study reports differences among the increase in skin fold thickness and among the number of reactors to the SIT and CIT tests when administered to the cervical and scapular inoculation sites, depending on the epidemiological situation of the herd. A poor or slight agreement in the number of reactors according to whether the test was carried out on the cervical or scapular sites was additionally observed, with the exception of the CIT test in the high-prevalence herd. These results highlight the importance of skin test standardization in goats and paves the way toward optimizing the intradermal tuberculin tests carried out on this species.

In the high prevalence herd, significantly greater reactions (in mm) were observed on the cervical site when compared to the scapular site. It is possible to hypothesize that this higher reactivity of the cervical site when compared to the scapular site could be related to the closer proximity to regional lymph nodes (cervical, submandibular, retropharyngeal, and parotid), which are in turn associated with the entrance route of TB infection (40, 41). Another reason could be the nature of the different areas of the skin on goats, such as their thickness (42, 43). Several studies have been carried out with cattle in order to evaluate the use of their necks, shoulders, and upper and lower sides for skin tests. These studies have determined that these various skin sites differ as regards sensitivity to the intradermal PPDs inoculation, with the neck being the most sensitive site (14, 19, 26, 28, 44, 45). In this respect, with regard to other species, a previous study performed with alpacas demonstrated that the probability of detecting TB-infected alpacas using the SIT test was not significantly affected by the bovine PPD inoculation site (axillary, prescapular, or cervical regions) (29). A similar finding was described in a recent study carried out with red deer using avian PPD on a TB-free farm with precedents of non-tuberculous mycobacteria infection (30). Nevertheless, the study in question evaluated two different avian PPD inoculation sites, both of which were located in the neck (anterior and caudal). In our study, there were no significant differences in the number of reactors to the SIT test when it was administered on both the cervical and scapular sites, although a higher number of reactors to the CIT test were detected on the cervical site in the high prevalence herd when using the standard interpretation. These results were slightly different from those obtained in a previous study of TB-infected cattle, in which a significantly higher number of reactors to the SIT test was observed after the bovine PPD injection on the cervical site when compared to prescapular site, thus suggesting a higher sensitivity of the intradermal test in the case of the cervical area (28). In this respect, we observed a higher occurrence of local clinical signs on the cervical site than on the scapular site, signifying that the number of reactors increased in comparison to when only the increase in skinfold thickness was taken into account.

In the low prevalence herd, the proportion of reactors to SIT test when using the severe interpretation criteria was significantly higher on the scapular site. However, most of the animals that were positive only on the scapular site were negative to the CIT test, IGRA, and P22 ELISA. Bearing in mind the low prevalence previously observed in this herd and the lack of agreement with the CIT test, IGRA, and P22 ELISA, the positives reactions on the scapular site could be related to a reduced specificity when compared to the cervical site in goats. In this respect, Casal et al. established that the probability of detecting a reactor to a skin test as regards any specific inoculation site was strongly associated with the prevalence, since higher values were observed in herds with a high proportion of reactors (28). It is also important to note that the animals from the low prevalence herd were the youngest in the study (data not shown) and had been vaccinated against MAP at 6 months of age (maximum age allowed). In this respect, it has been demonstrated that MAP vaccination may cause the onset of false-positive reactors to the SIT test if applied before 12 months post-vaccination in a TB-free/PTB-vaccinated herd (46). We did not, however, observe an association between age and increase in skinfold thickness after the avian and bovine PPD injections in our study in this herd (data not shown).

No positive reactors to the intradermal tests were observed when using the recommended standard interpretation in the TB-free herd, which proved to have an excellent specificity. Although 7 out of 119 animals were detected as being positive in the case of the cervical site when using the severe interpretation of the SIT test, this criterion is not recommended in TB-free and MAP-vaccinated herds, particularly in the first months after vaccination. Moreover, none of these positive reactors to the severe SIT test were positive to IGRA or the P22 ELISA, regardless of the cut-off point used. These results are supported by previous studies in TB-free cattle, in which greater skin responsiveness was observed in the anterior neck when compared with the area nearer to the shoulder (19, 45).

The CIT test was used to evaluate the effect of the inoculation site after the avian PPD inoculation in MAP vaccination circumstances. In our study, all three herds were vaccinated against MAP, which could be associated with the reactivity to the avian PPD observed (47). Differences in the number of reactors to the SIT and CIT tests according to the locations in which they were administered were, therefore, associated with the avian reactors. A significantly higher number of reactors was observed when using the standard interpretation of the CIT test in the neck compared to scapula in the high prevalence herd. Previous studies with cattle have determined that the neck is the most sensitive area in which to carry out the CIT test when compared to other areas, such as the shoulder, back, and sides (14, 44). In this respect, Good and collaborators performed a study with Irish cattle in which the cattle received two bovine and two avian PPD inoculations on different sites of the neck (19). However, the scapular area was not evaluated, signifying that it is not possible to make comparisons between this and the present study.

TB compatible lesions were previously observed in animals from the low and high prevalence herds of the present study at the slaughterhouse, and were confirmed as TB-infected (*M. bovis* SB0121) by means of bacteriological culture, although the study population was not subjected to individual post-mortem examination. The use of *in vitro* cell-based and humoral tests, such as IGRA or the P22 ELISA, therefore, makes it possible to obtain better knowledge of the immunological status of the animals. It is necessary to state that none of the goats from the low prevalence and TB-free herd that reacted only on one of both sites (severe interpretation in the TB-free herd) attained a positive result to IGRA, and few were positive to the P22 ELISA. In this respect, a recent study showed that the sensitivity of IGRA is higher than that observed for the SIT/CIT test in goats (13). Moreover, IGRA attained an excellent specificity in the TB-free herd. However, with regard to the P22 ELISA, the 100 E% cut-off point designed to increase the sensitivity was to the detriment of a certain specificity. In this context, a higher cut-off point (150 E%) has been demonstrated to attain an increase in specificity in goats (38). In addition, the MAP vaccination or infection of the herds studied could have had an influence on the serological results obtained. Recent studies have evidenced a lower specificity of the P22 ELISA in MAP vaccinated goats than in non-vaccinated goats, thus indicating interference owing to MAP vaccination (38, 46). Furthermore, the parallel interpretation of skin tests and IGRA detected less reactors than the combination with the P22 ELISA. These results support those obtained in previous studies with cattle and goats, in which the highest number of reactors were detected through the combination of the SIT test and the P22 ELISA (48–50).

The scapular site is more accessible in dairy goats during milking sessions and, therefore, makes the intradermal test easier when compared to that carried out on the neck, since the cervical inoculation entails the immobilization of the animals in order to avoid movements that may hamper the correct inoculation and reading. However, the skin test can be carried out on the cervical or scapular sites without logistical difficulties, and both are recommended in the “Intradermal Tuberculin Skin Test Protocol in caprine animals” of the European Union Reference Laboratory for Bovine Tuberculosis (51). Nevertheless, this procedure would alter if the performance of skin tests on a particular PPD inoculation site (cervical or scapular) was demonstrated to be significantly better in further studies. Most of the previous studies with goats concerning diagnosis have, to date, reported the use of the cervical test (13).

In summary, the present study has, for the first time, evaluated whether the PPD inoculation site has an effect on the results of the SIT and CIT tests in goats. The results obtained did not show a clear association between the PPD injection site and the diagnostic outcome, which differed depending on the status of the herd. These results do not, therefore, make it possible to conclude whether the cervical or the scapular inoculation sites could improve the performance of the SIT and CIT tests. However, the greater increase in skinfold thickness observed on the cervical site in the high prevalence herd suggests that this location could improve sensitivity without significantly compromising specificity, as observed in the TB-free herd. In fact, significantly more reactors to the CIT test when administered on the cervical site when compared to the scapular site were detected in the high prevalence herd.

## Data Availability Statement

The original contributions presented in the study are included in the article/supplementary material, further inquiries can be directed to the corresponding author/s.

## Ethics Statement

Ethical review and approval was not required for the animal study because the animals included in the study were not experimental animals. All handling and sampling procedures were carried out in accordance with local and Spanish legislation (Royal Decree 727/2011). Written informed consent was obtained from the owners for the participation of their animals in this study.

## Author Contributions

JO, ÁR, and JB wrote the manuscript, performed the literature search, and designed the figures. JO, JS-C, ML, and ÁR performed the experiments. JÁ, JO, JI-L, JB, LD, ÁR, LJ, JS, and BR interpreted the data. All authors reviewed and approved the manuscript.

## Conflict of Interest

The authors declare that the research was conducted in the absence of any commercial or financial relationships that could be construed as a potential conflict of interest.

## Publisher's Note

All claims expressed in this article are solely those of the authors and do not necessarily represent those of their affiliated organizations, or those of the publisher, the editors and the reviewers. Any product that may be evaluated in this article, or claim that may be made by its manufacturer, is not guaranteed or endorsed by the publisher.
